# Presence of antibody-dependent cellular cytotoxicity (ADCC) against SARS-CoV-2 in COVID-19 plasma

**DOI:** 10.1371/journal.pone.0247640

**Published:** 2021-03-04

**Authors:** For Yue Tso, Salum J. Lidenge, Lisa K. Poppe, Phoebe B. Peña, Sara R. Privatt, Sydney J. Bennett, John R. Ngowi, Julius Mwaiselage, Michael Belshan, Jacob A. Siedlik, Morgan A. Raine, Juan B. Ochoa, Julia Garcia-Diaz, Bobby Nossaman, Lyndsey Buckner, W. Mark Roberts, Matthew J. Dean, Augusto C. Ochoa, John T. West, Charles Wood

**Affiliations:** 1 Nebraska Center for Virology, University of Nebraska-Lincoln, Lincoln, NE, United States of America; 2 School of Biological Sciences, University of Nebraska-Lincoln, Lincoln, NE, United States of America; 3 Ocean Road Cancer Institute, Dar es Salaam, Tanzania; 4 Muhimbili University of Health and Allied Sciences, Dar es Salaam, Tanzania; 5 Department of Medical Microbiology & Immunology, Creighton University, Omaha, NE, United States of America; 6 Department of Exercise Science and Pre-Health Professions, Creighton University, Omaha, NE, United States of America; 7 Department of Surgery, Ochsner Medical Center, New Orleans, LA, United States of America; 8 Department of Internal Medicine Ochsner Medical Center, New Orleans, LA, United States of America; 9 Louisiana State University Cancer Center, New Orleans, LA, United States of America; 10 Department of Pediatrics LSU Health, New Orleans, LA, United States of America; 11 Department of Biochemistry, University of Nebraska-Lincoln, Lincoln, NE, United States of America; National Institute of Child Health and Human Development, UNITED STATES

## Abstract

**Background:**

Neutralizing-antibody (nAb) is the major focus of most ongoing COVID-19 vaccine trials. However, nAb response against SARS-CoV-2, when present, decays rapidly. Given the myriad roles of antibodies in immune responses, it is possible that antibodies could also mediate protection against SARS-CoV-2 via effector mechanisms such as antibody-dependent cellular cytotoxicity (ADCC), which we sought to explore here.

**Methods:**

Plasma of 3 uninfected controls and 20 subjects exposed to, or recovering from, SARS-CoV-2 infection were collected from U.S. and sub-Saharan Africa. Immunofluorescence assay was used to detect the presence of SARS-CoV-2 specific IgG antibodies in the plasma samples. SARS-CoV-2 specific neutralizing capability of these plasmas was assessed with SARS-CoV-2 spike pseudotyped virus. ADCC activity was assessed with a calcein release assay.

**Results:**

SARS-CoV-2 specific IgG antibodies were detected in all COVID-19 subjects studied. All but three COVID-19 subjects contained nAb at high potency (>80% neutralization). Plasma from 19/20 of COVID-19 subjects also demonstrated strong ADCC activity against SARS-CoV-2 spike glycoprotein, including two individuals without nAb against SARS-CoV-2.

**Conclusion:**

Both neutralizing and non-neutralizing COVID-19 plasmas can mediate ADCC. Our findings argue that evaluation of potential vaccines against SARS-CoV-2 should include investigation of the magnitude and durability of ADCC, in addition to nAb.

## Introduction

The coronavirus disease of 2019 (COVID-19) pandemic is caused by the novel SARS-CoV-2 virus [1, 2]. According to the latest report from the Johns Hopkins Coronavirus Resource Center, as of Feb 5, 2021, SARS-CoV-2 has infected >100 million individuals worldwide, and >26 million in the U.S. alone, leading to >450 thousand deaths [3]. With a wide variety of vaccine candidates currently in various stages of clinical trials worldwide, it is important to consider what vaccine correlates are likely to promote responses of sufficient magnitude and durability to impart protection. Antibody responses develop against SARS-CoV-2 during the infection in many subjects tend to increase over the course of disease and correlate with viral RNA titer [4]. Neutralizing antibody (nAb) responses have been shown to preferentially target the receptor binding domain (RBD) of the SARS-CoV-2 spike glycoprotein (S), but the levels of nAb were variable in infected subjects and can undergo fairly rapid decay kinetics [5, 6]. Other non-RBD-specific Ab which target the SARS-CoV-2 S could be less apt to neutralization, but nevertheless have important roles in viral control by coupling adaptive humoral responses to natural killer (NK) cells through the mechanism of Ab-dependent cellular cytotoxicity (ADCC).

Convalescent plasma has been used successfully against other infectious diseases such as influenza and SARS and remains among the potential COVID-19 therapies producing efficacy against COVID-19 in several small-scale studies [7–10]. Neutralization is considered a mechanism of action for SARS-CoV-2 convalescent plasma. Additionally, other non-neutralizing antibody-dependent effector mechanisms such as antibody-dependent cellular phagocytosis (ADCP), complement-dependent cytotoxicity (CDC) and antibody-dependent cellular cytotoxicity (ADCC) have been shown to play a role in protection against other viruses [11–14]. For ADCC, NK cells recognize and bind to Ab opsonized (targeted) cells using their FcγRIII receptor, CD16, leading to perforin and granzyme degranulation-mediated cytotoxicity of the infected target cells. Since other humoral effector mechanisms have not been investigated for efficacy in SARS-CoV-2 infection, we sought to explore whether ADCC was evident in plasma from recovered or recovering COVID-19 patients in this study.

## Materials and methods

### Study cohort

This study comprised of 23 consenting subjects, ≥18 years of age and of both genders from U.S. and Sub-Saharan Africa (SSA). The SSA samples included 2 plasma samples from confirmed COVID-19 individuals (SSA1 and SSA2), 1 COVID-19 exposed but unconfirmed by SARS-CoV-2 RT-PCR individual (SSA3) and 3 pre-pandemic voluntary blood donor plasma samples (N1, N2 and N3). The pre-pandemic samples were collected in SSA between March and May of 2019. Seventeen COVID-19 plasma samples (US1 to US17) were obtained from U.S.. All COVID-19 diagnoses were determined by local health providers with RT-PCR of SARS-CoV-2 in the buccal and/or nasopharyngeal swabs. All study procedures were approved by the institutional review board at the University of Nebraska–Lincoln.

### Cell lines

HEK-293T cells (CRL-3216, ATCC, Manassas, VA, USA) were cultured in Dulbecco’s modified Eagle medium (DMEM) with 10% fetal bovine serum (FBS) and 1% penicillin–streptomycin (P/S).

HEK-293T-hACE2 cells (HEK-293T cells expressing the human angiotensin-converting enzyme 2) (NR-52511, BEI Resources, Manassas, VA, USA) were cultured in DMEM with 10% FBS and 1% P/S.

NK92.05-CD16-176V, a natural killer cell line engineered to express the high affinity FcγRIII (generously provided by Dr. Kerry Campbell at Fox Chase Cancer Center) were maintained in αMEM complete media: MEM (M0644, Sigma, Burlington, MA, USA) supplemented with 2.2g/L sodium bicarbonate (25080094, ThermoFisher Scientific, Waltham, MA), 0.1mM 2-mercaptoethanol (31350010, ThermoFisher Scientific, Waltham, MA), 2mM L-glutamine (25005CI, Corning, NY, USA), 0.2mM myo-inositol (I5125, Sigma, Burlington, MA, USA), 0.02mM folic acid (F7876, Sigma, Burlington, MA, USA), 1% non-essential amino acids (11140050, ThermoFisher Scientific, Waltham, MA), 1% sodium pyruvate (11360070, ThermoFisher Scientific, Waltham, MA), 1% P/S, 12.5% FBS, and 12.5% horse serum (H1138, Sigma, Burlington, MA, USA). The cells were passaged every 4 days in the presence of 2.5–5% freshly thawed J558L supernatant (see human IL-2 production).

J558L Hu-IL-2 cells, a mouse myeloma cell line that expresses human IL-2 (provided by Dr. Kerry Campbell at Fox Chase Cancer Center) were cultured in RPMI media with 10% FBS, 1% P/S, 2mM L-glutamine, 1% sodium pyruvate, 0.1mM 2-mercapotethanol, and 1% HEPES (25060CI, ThermoFisher Scientific, Waltham, MA). All cells were maintained in 5% CO_2_ incubator at 37°C.

### Human IL-2 production

J558L Hu-IL-2 cells were used to produce human IL-2 as a growth supplement for NK92.05-CD16-176V cells. The cells were initiated in 10 ml culture media (see above) in a T25 flask. The day after thaw, the culture was transferred to a T75 flask and brought to a total volume of 30 ml. The cells were then expanded every 2–3 days until the desired volume was achieved. The cells were allowed to grow for about one week, until the media turned yellow. The culture was then centrifuged for 3 minutes at 1300 rpm. Supernatant was 0.22 μm filtered, aliquoted and frozen at -80°C.

### Cells expressing SARS-CoV-2 spike and nucleocapsid proteins

Cells expressing either the SARS-CoV-2 spike or nucleocapsid proteins were generated for use in the immunofluorescence assay (IFA) and as target cells in the ADCC assay. At 24-hours before transfection, 8 x 10^5^ HEK-293T cells per well were seeded into a 6-well plate with DMEM, 20% FBS without P/S. The cells in each well were transfected with 2 μg of either SARS-CoV-2 spike mammalian expression plasmid pcDNA3.1-SARS2-S (a gift from Dr. Fang Li, 145032, Addgene, Watertown, MA, USA) [15] or SARS-CoV-2 nucleocapsid mammalian expression plasmid pGBW-m4134903 (a gift from Ginkgo Bioworks, 151951, Addgene, Watertown, MA, USA), using Fugene 6 (E2692, Promega, Madison, WI, USA) in Opti-MEM reduced serum medium (31985070, ThermoFisher Scientific, Waltham, MA). The transfected cells were incubated at 37°C in a 5% CO_2_ incubator for 48-hours.

### Immunofluorescence assay (IFA) against SARS-CoV-2

To generate the microscopy slides for IFA, at 48-hours post-transfection, the transfected HEK-293T cells expressing either the SARS-CoV-2 spike or nucleocapsid proteins were harvested without trypsin, fixed with 4% PFA and seeded onto 12-well polytetrafluoroethylene (PTFE) printed slides (6342505, Electron Microscopy Sciences, Hatfield, PA, USA). Each well contained either mock, spike or nucleocapsid transfected cells. The cells were then permeabilized with 0.3% H_2_O_2_ methanol solution, washed with 1X PBS, air-dried and stored at -80°C. The donor plasmas were first heat-inactivated at 56°C for 1 hour, spun at 12,000 x g for 5 minutes to remove any debris or aggregates, diluted at 1:20 with 1X PBS containing 0.1% Tween-20 and incubated at room temperature for 30 minutes. The IFA slides were thawed and incubated with 1X PBS containing 0.1% Tween-20 for 30 minutes at 37°C in a humidity chamber. Fifteen microliters of the diluted plasmas were then added onto each well and incubated for 1 hour at 37°C in a humidity chamber. After washing with 1X PBS, the secondary mouse monoclonal anti-human IgG antibody (CRL-1786, ATCC, Manassas, VA, USA) was added and incubated for 1 hour at 37°C in a humidity chamber. After washings with 1X PBS, the slides were incubated with the tertiary CY2 conjugated donkey anti-mouse IgG (715225150, Jackson ImmunoResearch, West Grove, PA, USA) for 1 hour at 37°C in a humidity chamber. Finally, the slides were washed, stained with 0.004% Evans Blue solution for 30 seconds and washed to remove excess staining solution. The slides were air-dried and protected by a cover slip with Fluoromount Aqueous Mounting Medium (F4680, Sigma, Burlington, MA, USA). The slides were examined by three independent readers using Nikon Eclipse 50i fluorescence microscope. A sample was only considered positive if the results from at least two readers concurred.

### Neutralization assay

SARS-CoV-2 spike glycoprotein pseudotyped lentivirus were generated by co-transfection of HEK-293T cells with SARS-CoV-2 spike mammalian expression plasmid (pcDNA3.1-SARS2-S, a gift from Dr. Fang Li, 145032, Addgene, Watertown, MA, USA) [15], 3^rd^ generation lentiviral plasmid encoding EGFP (FUGW, a gift from Dr. David Baltimore, 14883, Addgene, Watertown, MA, USA) [16] and the packaging plasmid (psPAX2, a gift from Dr. Didier Trono, 12260, Addgene, Watertown, MA, USA). The culture supernatant containing the pseudotyped virus was collected at 72-hours post-transfection and concentrated by ultracentrifugation.

At 24-hours before the neutralization assay, 1 x 10^4^ HEK-293T-hACE2 cells per well were seeded into a 96-well plate. The donor plasmas were heat inactivated at 56°C for 1 hour and diluted at 1:40 with culture medium and 25 μl of the SARS-CoV-2 Spike pseudotyped virus for a total volume of 200 μl per well. The plasma-virus mixtures were then incubated at 37°C for 1 hour. The old culture medium of the pre-plated HEK-293T-hACE2 cells was then replaced with the plasma-virus mixture, spun at 400 x g for 20 minutes and incubated at 37°C in a 5% CO_2_ incubator for 72 hours. The level of infection was determined by quantification of the GFP signal using BD Accuri C6 Plus flow cytometer (BD Biosciences, San Jose, CA, USA) and flow data analyzed by FlowJo software (BD Life Sciences, San Jose, CA, USA). Each plasma sample was tested in triplicate. Each set of experiment contained mock-only cells and virus-only cells. The percent GFP in mock cells was subtracted from all other samples, including virus-only cells. To calculate the final percent neutralization, the following equation was used: (Virus only–Sample)/Virus only x 100%. The data were then plotted and statistical analysis was conducted using GraphPad Prism version 5.05 (GraphPad Software, San Diego, CA, USA).

### Antibody-dependent cellular cytotoxicity (ADCC) assay

ADCC activity was assessed with a calcein release assay [17, 18]. Target cells (HEK-293T, either mock transfected or expressing SARS-CoV-2 spike) were labeled with 2 μg/ml Calcein-AM (C3099, ThermoFisher Scientific, Waltham, MA) at a concentration of 10^6^ cells/ml for 30 minutes at 37°C, 5% CO_2_. After labeling, excess dye was removed by washing the cells twice with DMEM plus 10% FBS and cells were resuspended at a final concentration of 10^6^ cells/ml. The labeled target cells (10^5^ cells) were aliquoted at 100 μl per well into a 96-well v-bottom plate. Sample plasma at a final dilution of 1:50 (4 μl) were added to each experimental well and allowed to incubate at room temperature for 15 minutes while the effector cells were prepared. NK92.05-CD16-176V effector cells were resuspended at 5 x 10^6^ cells/ml in αMEM complete media and 100 μl (5 x 10^5^ cells) were added to all wells, except the spontaneous and maximum release control wells (Target cells only), for an effector to target ratio of 5:1. Maximum release was achieved through the addition of 104 μl of 0.1% Triton-X-100, while 104 μl of αMEM complete media were added to spontaneous release control wells. The 96-well v-bottom plate was then spun at 100 x g for 2 minutes to increase cell interactions and incubated at 37°C, 5% CO_2_ for 4 hours. Following incubation, the wells were mixed via gentle pipetting, and spun at 400 x g for 2 minutes to pellet the cells and debris. The supernatant (150 μl) was transferred to a black-walled 96-well clear bottom plate and fluorescence was determined with Victor^3^V plate reader (PerkinElmer, Waltham, MA, USA).

Each plate contained the following controls: target cells only (spontaneous release); target cells plus Trition-X-100 (maximum release); target cells and effectors, no plasma (TE). To calculate the percent ADCC activity, this formula was used: [(Experimental-Spontaneous)—(TE-Spontaneous)]/(Maximum-Spontaneous)*100%. Data is presented as the change in ADCC (ΔADCC), where ADCC against mock is subtracted from ADCC against spike expressing cells (i.e. ADCC of spike–ADCC of mock). All samples were tested in quintuplicate and data shown is the mean with standard deviation of the centroid three replicates. The data were then plotted and statistical analysis (Mann Whitney) was performed using GraphPad Prism version 5.05 (GraphPad Software, San Diego, CA, USA).

## Results

To evaluate humoral neutralizing and ADCC responses against SARS-CoV-2 and to investigate potential relationships between the two, we obtained plasmas from 18 confirmed COVID-19 symptomatic individuals, 1 confirmed COVID-19 asymptomatic individual, 1 presumed highly exposed but asymptomatic SARS-CoV-2 seropositive individual and 3 SARS-CoV-2 seronegative blood donors from the U.S. and sub-Saharan Africa (SSA) (Table 1). Among the 17 U.S. COVID-19 samples, 5 individuals (US1, US2, US3, US16 and US17) had recovered from COVID-19 when the plasma samples were collected. The remaining 12 U.S. samples were individuals with severe COVID-19 infection that required hospitalization. Two RT-PCR confirmed cases SSA1 and SSA2, symptomatic and asymptomatic, respectively, as well as an exposed but non-RT-PCR confirmed seropositive individual (SSA3) (cohabitating household member of SSA2 as well as a confirmed COVID-19 spouse) were from SSA. These individuals all recovered from COVID-19. The three negative control cases (N1, N2 and N3) were pre-pandemic healthy blood donors from SSA.

**Table 1:** Study cohort information.

Sample ID	Area of Origin	Age	Gender	COVID-19 diagnosis by RT-PCR	Symptomatic/Asymptomatic	Disease Severity
US1	US	32	Male	Confirmed	Symptomatic	Convalescent
US2	US	35	Male	Confirmed	Symptomatic	Convalescent
US3	US	22	Female	Confirmed	Symptomatic	Convalescent
US4	US	61	Female	Confirmed	Symptomatic	Hospitalized
US5	US	76	Male	Confirmed	Symptomatic	Hospitalized
US6	US	51	Female	Confirmed	Symptomatic	Hospitalized
US7	US	60	Male	Confirmed	Symptomatic	Hospitalized
US8	US	61	Female	Confirmed	Symptomatic	Hospitalized
US9	US	81	Male	Confirmed	Symptomatic	Hospitalized
US10	US	79	Male	Confirmed	Symptomatic	Hospitalized
US11	US	72	Male	Confirmed	Symptomatic	Hospitalized
US12	US	59	Male	Confirmed	Symptomatic	Hospitalized
US13	US	76	Female	Confirmed	Symptomatic	Hospitalized
US14	US	79	Female	Confirmed	Symptomatic	Hospitalized
US15	US	85	Male	Confirmed	Symptomatic	Hospitalized
US16	US	31	Female	Confirmed	Symptomatic	Convalescent
US17	US	59	Female	Confirmed	Symptomatic	Convalescent
SSA1	SSA	26	Male	Confirmed	Symptomatic	Mild
SSA2	SSA	31	Female	Confirmed	Asymptomatic	N/A
SSA3	SSA	32	Male	N/A	Asymptomatic	N/A
*N1 to N3	SSA	N/A	N/A	N/A	N/A	N/A

“*” denotes pre-pandemic negative control plasmas.

“US” denotes United States of America.

“SSA” denotes sub-Saharan Africa.

N/A denotes not-applicable or not-available.

**Fig 1:**
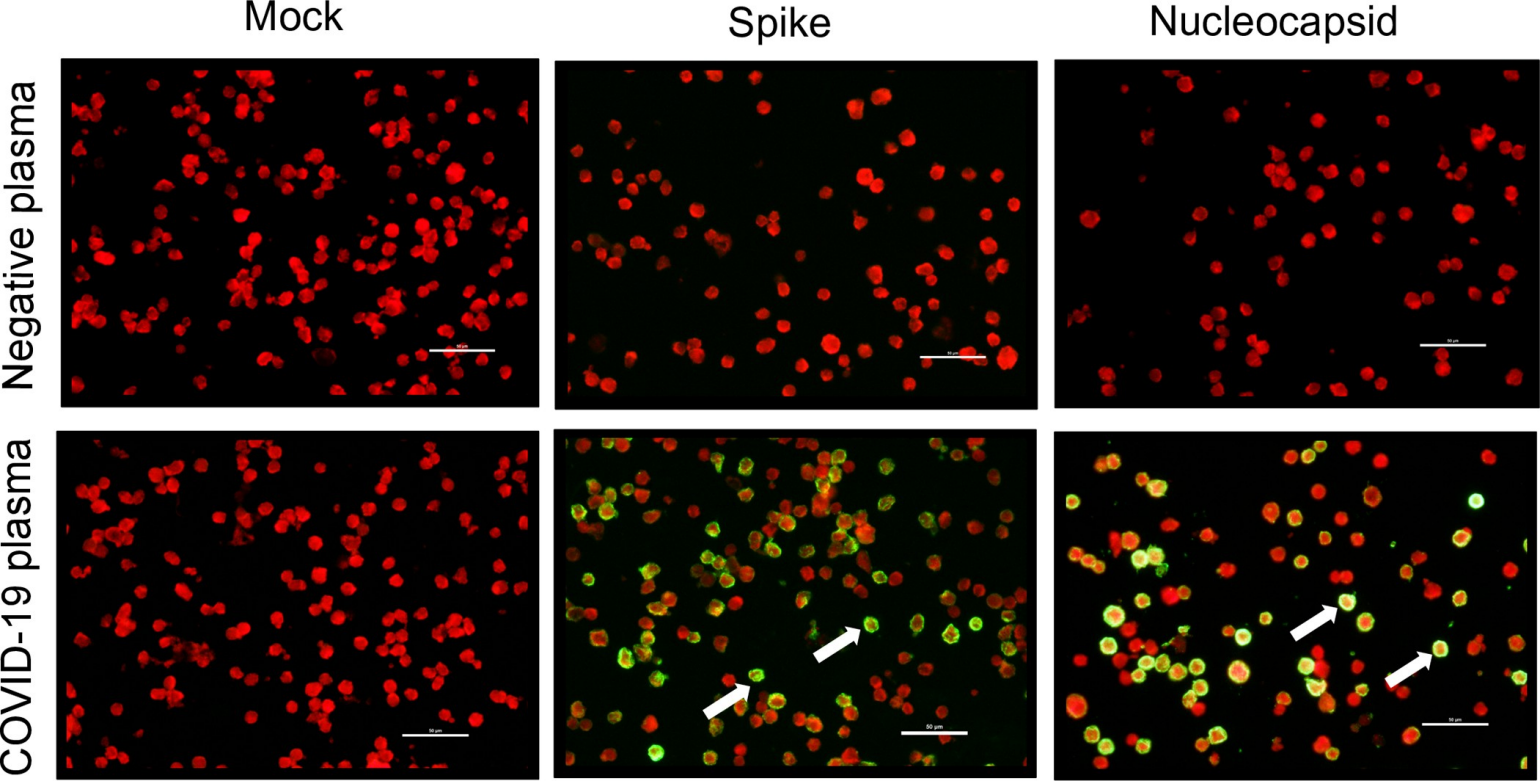
Immunofluorescence assay (IFA) against SARS-CoV-2 proteins. Representative pictures of IFA against either the mock, SARS-CoV-2 spike or nucleocapsid expressing HEK-293T cells. The upper row shows IFA with negative control plasma collected before the COVID-19 pandemic. The lower row shows IFA with COVID-19 plasma, where strong green color positive cells (indicated by white arrows) were only observed in cells expressing either SARS-CoV-2 spike or nucleocapsid proteins. The lack of green color positive cells with negative control plasma and mock cells demonstrated the specificity of the IFA. All pictures were taken at 20X magnification with Nikon Eclipse 50i fluorescence microscope. The white scale bars indicate 50 μm.

To determine if plasma from these COVID-19 cases contained SARS-CoV-2 specific IgG antibodies, we developed an in-house immunofluorescence assay (IFA) using HEK293T cells expressing either SARS-CoV-2 S or nucleocapsid (N) proteins [19]. The assay detects binding of SARS-CoV-2 specific IgG in the plasma to the viral proteins expressed in these cells. After the addition of a secondary anti-human IgG antibody followed by a fluorescently tagged tertiary antibody, SARS-CoV-2 positive plasma produced a green color in epifluorescence microscopy (Fig 1). The expression of SARS-CoV-2 S and N proteins in HEK293T cells, 57.6% and 58.4% respectively, were assessed via IFA with convalescent COVID-19 plasma. Using this method, we detected strong IgG antibody responses against the SARS-CoV-2 S glycoprotein in all COVID-19 individuals, and against the N protein in 18/20 COVID-19 individuals. Surprisingly, COVID-19 individuals US13 and SSA3 only showed strong responses against the S glycoprotein, but no response against N. This result reinforces the concept that SSA3 was sufficiently exposed to SARS-CoV-2 to generate an Ab response to a viral surface glycoprotein, without generating a N protein response or exhibiting symptoms. No fluorescent staining (green) was observed against SARS-CoV-2 S- and N- expressing cells by negative control plasmas. Likewise, no staining (green) was evident when SARS-CoV-2 plasma was applied to mock transfected cells in parallel (Fig 1).

**Fig 2:**
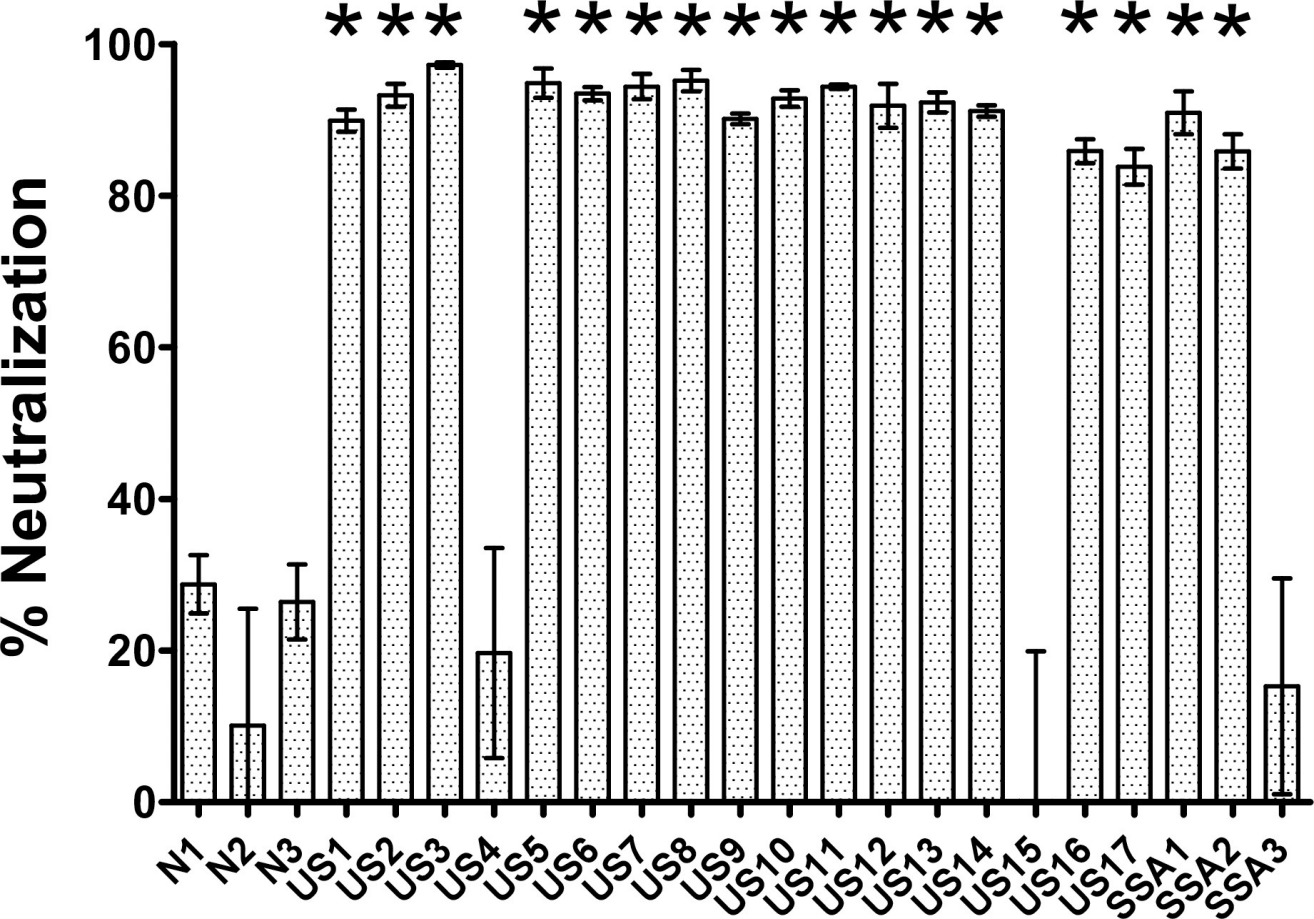
SARS-CoV-2 spike pseudotyped virus neutralization assay. SARS-CoV-2 spike pseudotyped lentivirus virus encoding EGFP were tested against negative control pre-pandemic plasmas (N1, N2 and N3) and COVID-19 plasmas (US1 to US17 and SSA1 to SSA3) at 1:40 plasma dilution. At 72-hours post-infection, percentage of GFP positive cells were quantified with BD Accuri C6 Plus flow cytometer. The mean of triplicate wells was shown with error bars representing SEM. P-values were calculated via one-way ANOVA and “*” denotes p < 0.05 relative to negative control plasmas.

Since the detection of SARS-CoV-2 specific IgG does not guarantee the presence of neutralizing antibodies, we used a SARS-CoV-2 S-pseudotyped virus neutralization assay to evaluate the presence of such antibodies. We detected significantly elevated neutralization at 84–97% (p<0.05) in 17/20 COVID-19 plasmas compared to plasma from negative controls (Fig 2). This finding is consistent with other studies showing nAb in the majority of COVID-19 infected individuals [20]. It was surprising, however, that there was no significant nAb activity in the plasma from COVID-19 individuals US4, US15 and SSA3, despite the fact that they had detectable anti-S glycoprotein antibodies. It is possible that at the time of sample collection these individuals were in early stages of infection and had experienced insufficient time or viremia to develop detectable levels of nAb. In addition, these individuals may have other factors that weaken their humoral responses. Given that SSA3 remained asymptomatic despite two sources of household exposure, it is also plausible that T-cell responses, or alternatively, non-neutralizing Ab effector responses, such as ADCC (targeted against the S glycoprotein), may have provided protection against symptomatic COVID-19.

**Fig 3:**
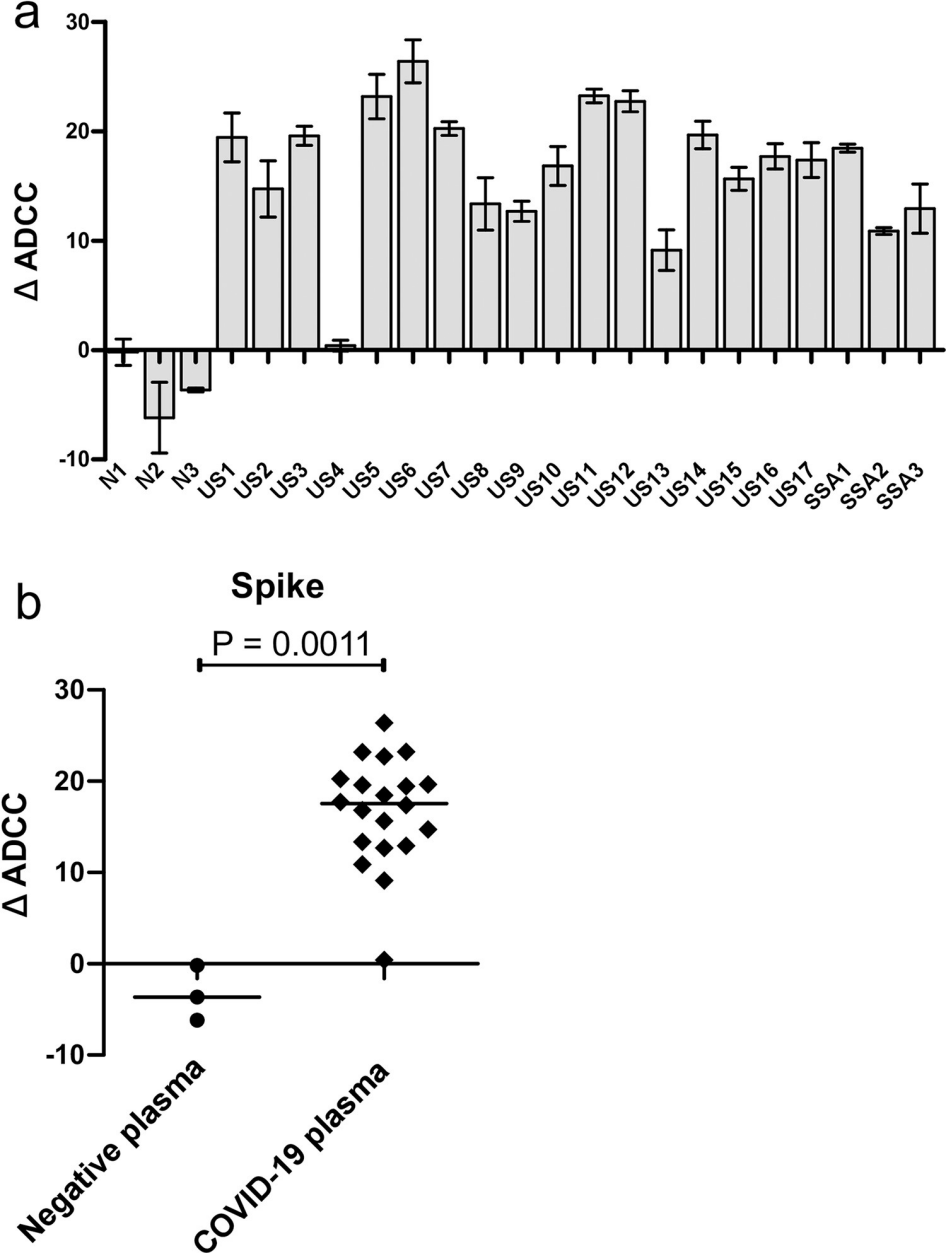
Antibody-dependent cellular cytotoxicity (ADCC) assay. The ADCC activity of COVID-19 plasmas were tested against HEK-293T cells expressing SARS-CoV-2 spike protein, which served as the target cells. After incubation with plasma, Calcein-AM labeled target cells were incubated with NK cells (NK92.05-CD16-176V) which served as the effector cells. The amount of fluorescent calcein released into the medium was then measured with Victor^3^V plate reader. A) changes in ADCC (ΔADCC) activity against the spike protein, relative to their respective activity against mock cells. N denotes pre-pandemic negative control plasmas. US and SSA denotes COVID-19 samples from USA and sub-Saharan Africa, respectively. B) Comparison of ΔADCC against the spike protein between COVID-19 and negative control plasmas. P-values were calculated via Mann Whitney test.

To test for non-neutralizing responses, we performed a calcein-release ADCC assay with NK92.05-CD16-176V effector cells to measure SARS-CoV-2 specific responses against mock-transfected cells and cells expressing S protein (Fig 3). Target cells for ADCC were produced by transfecting HEK-293T cells with mammalian CMV-promoter SARS-CoV-2 S expression plasmid and 57.6% of the cells express the S protein after transfection as shown in Fig 1. The target cells were then labeled with non-fluorescent Calcein-AM, a dye that is intracellularly converted to green fluorescent calcein. The NK92.05-CD16-176V effector cells will recognized these dye-labeled and Ab decorated antigen expressing target cells via its FcγRIII receptor that binds to the Fc region of the bound antibody. This recognition activates the NK cell which then disrupts the plasma membrane of target cells and release calcein into the culture supernatant, where it was quantified by a fluorescent plate reader. We found that plasma in 19/20 SARS-CoV-2 infected/exposed individuals induced ADCC activity against S glycoprotein-expressing targets (Fig 3A) that was significantly higher (P = 0.0011) compared to negative controls (Fig 3B). All individuals with nAb also demonstrated ADCC activity. Of the three individuals without nAb, sample US4 was the only individual who had SARS-CoV-2 spike specific antibodies that were non-neutralizing and unable to induce ADCC activity. The reason for the absence of ADCC activity in US4 is not clear, even though there are antibodies that bind to both spike and nucleocapsid proteins. One possible explanation is the lack of anti-SARS-CoV-2 antibody subclasses that can bind the FcγRIII receptor with high affinity to mediate ADCC in our assay [21]. The remaining two individuals, US15 and SSA3 had SARS-CoV-2 spike specific antibodies as detected by IFA but lacked nAb. Yet, their plasma was still able to induce significant ADCC activity against the S-expressing target cells, strongly indicating that non-neutralizing antibodies might play a role against SARS-CoV-2 via ADCC.

## Discussion

Our study demonstrated that specific SARS-CoV-2 S glycoprotein targeting antibodies from COVID-19 plasma can induce ADCC killing via NK cells *in-vitro*. Although speculation as to the presence of ADCC against SARS-CoV-2 has been reported, there has been little direct experimental evidence [22, 23]. Several studies showed human and murine mAb against SARS-CoV displayed cross-reactive ADCC responses against SARS-CoV-2, but no study has examined COVID-19 plasma for its capacity to directly mediate ADCC [24, 25]. Despite our findings and those in previous reports, whether SARS-CoV-2 specific ADCC actually occurs *in-vivo*, will require further investigation with a larger sample size and functional testing of NK cells from the infected individuals in conjunction with autologous plasma. Several studies have suggested dysfunction and decreased number of NK cells in patients with severe COVID-19 disease, which may undermine the role that NK cells and ADCC play in disease recovery [26, 27]. Nevertheless, ADCC may still be an important factor in vaccine efficacy. Moreover, in addition to NK cells, other FcγRIII receptor-expressing cells, such as macrophages, neutrophils, and eosinophils, could also mediate ADCC. The detection of ADCC inducing-antibodies in COVID-19 individuals also supports the exploration of other antibody-dependent effector mechanisms, such as ADCP and CDC, and their role in COVID-19 pathogenesis and recovery.

The major implication of our findings is that efficacy of COVID-19 vaccine candidates should not be evaluated solely based on the level of nAb elicited, but rather the totality of SARS-CoV-2 specific humoral responses elicited. The potential contributions and durability of non-neutralizing antibody effector mechanisms need to be included in such assessments. Therefore, in addition to T-cell responses, a qualitative examination of other immune cells such as NK cells and macrophages should also be taken into consideration. Clearly, given the importance and urgency of developing an effective COVID-19 vaccine, a larger more in-depth study will be warranted to dissect the role of non-neutralizing Ab more completely in COVID-19 patients and vaccinated individuals.

## References

[1] Wu F, Zhao S, Yu B, Chen YM, Wang W, Song ZG, et al. A new coronavirus associated with human respiratory disease in China. Nature. 2020;579(7798):265–9. 10.1038/s41586-020-2008-3 32015508 PMC7094943

[2] Zhou P, Yang XL, Wang XG, Hu B, Zhang L, Zhang W, et al. A pneumonia outbreak associated with a new coronavirus of probable bat origin. Nature. 2020;579(7798):270–3. 10.1038/s41586-020-2012-7 32015507 PMC7095418

[3] Johns Hopkins Coronavirus Resource Center: Johns Hopkins University; 2020 [cited 2020. Available from: https://coronavirus.jhu.edu/.

[4] Chen Y, Li L. SARS-CoV-2: virus dynamics and host response. Lancet Infect Dis. 2020;20(5):515–6. 10.1016/S1473-3099(20)30235-8 32213336 PMC7156233

[5] Jiang S, Hillyer C, Du L. Neutralizing Antibodies against SARS-CoV-2 and Other Human Coronaviruses. Trends Immunol. 2020;41(5):355–9. 10.1016/j.it.2020.03.007 32249063 PMC7129017

[6] Ibarrondo FJ, Fulcher JA, Goodman-Meza D, Elliott J, Hofmann C, Hausner MA, et al. Rapid Decay of Anti-SARS-CoV-2 Antibodies in Persons with Mild Covid-19. N Engl J Med. 2020. 10.1056/NEJMc2025179 32706954 PMC7397184

[7] Duan K, Liu B, Li C, Zhang H, Yu T, Qu J, et al. Effectiveness of convalescent plasma therapy in severe COVID-19 patients. Proc Natl Acad Sci U S A. 2020;117(17):9490–6. 10.1073/pnas.2004168117 32253318 PMC7196837

[8] Shen C, Wang Z, Zhao F, Yang Y, Li J, Yuan J, et al. Treatment of 5 Critically Ill Patients With COVID-19 With Convalescent Plasma. JAMA. 2020. 10.1001/jama.2020.4783 PMC7101507 32219428

[9] Cheng Y, Wong R, Soo YO, Wong WS, Lee CK, Ng MH, et al. Use of convalescent plasma therapy in SARS patients in Hong Kong. Eur J Clin Microbiol Infect Dis. 2005;24(1):44–6. 10.1007/s10096-004-1271-9 15616839 PMC7088355

[10] Hung IF, To KK, Lee CK, Lee KL, Chan K, Yan WW, et al. Convalescent plasma treatment reduced mortality in patients with severe pandemic influenza A (H1N1) 2009 virus infection. Clin Infect Dis. 2011;52(4):447–56. 10.1093/cid/ciq106 21248066 PMC7531589

[11] Gorander S, Ekblad M, Bergstrom T, Liljeqvist JA. Anti-glycoprotein g antibodies of herpes simplex virus 2 contribute to complete protection after vaccination in mice and induce antibody-dependent cellular cytotoxicity and complement-mediated cytolysis. Viruses. 2014;6(11):4358–72. 10.3390/v6114358 25398047 PMC4246227

[12] Jenks JA, Goodwin ML, Permar SR. The Roles of Host and Viral Antibody Fc Receptors in Herpes Simplex Virus (HSV) and Human Cytomegalovirus (HCMV) Infections and Immunity. Front Immunol. 2019;10:2110. 10.3389/fimmu.2019.02110 31555298 PMC6742691

[13] Moraru M, Black LE, Muntasell A, Portero F, Lopez-Botet M, Reyburn HT, et al. NK Cell and Ig Interplay in Defense against Herpes Simplex Virus Type 1: Epistatic Interaction of CD16A and IgG1 Allotypes of Variable Affinities Modulates Antibody-Dependent Cellular Cytotoxicity and Susceptibility to Clinical Reactivation. J Immunol. 2015;195(4):1676–84. 10.4049/jimmunol.1500872 26179905

[14] Forthal DN, Finzi A. Antibody-dependent cellular cytotoxicity in HIV infection. AIDS. 2018;32(17):2439–51. 10.1097/QAD.0000000000002011 30234611 PMC6497078

[15] Shang J, Ye G, Shi K, Wan Y, Luo C, Aihara H, et al. Structural basis of receptor recognition by SARS-CoV-2. Nature. 2020;581(7807):221–4. 10.1038/s41586-020-2179-y 32225175 PMC7328981

[16] Lois C, Hong EJ, Pease S, Brown EJ, Baltimore D. Germline transmission and tissue-specific expression of transgenes delivered by lentiviral vectors. Science. 2002;295(5556):868–72. 10.1126/science.1067081 11786607

[17] Neri S, Mariani E, Meneghetti A, Cattini L, Facchini A. Calcein-acetyoxymethyl cytotoxicity assay: standardization of a method allowing additional analyses on recovered effector cells and supernatants. Clin Diagn Lab Immunol. 2001;8(6):1131–5. 10.1128/CDLI.8.6.1131-1135.2001 11687452 PMC96238

[18] Somanchi SS, McCulley KJ, Somanchi A, Chan LL, Lee DA. A Novel Method for Assessment of Natural Killer Cell Cytotoxicity Using Image Cytometry. PLoS One. 2015;10(10):e0141074. 10.1371/journal.pone.0141074 26492577 PMC4619620

[19] Tso FY, Lidenge SJ, Pena PB, Clegg AA, Ngowi JR, Mwaiselage J, et al. High prevalence of pre-existing serological cross-reactivity against SARS-CoV-2 in sub-Sahara Africa. Int J Infect Dis. 2020. 10.1016/j.ijid.2020.10.104 PMC7648883 33176202

[20] Robbiani DF, Gaebler C, Muecksch F, Lorenzi JCC, Wang Z, Cho A, et al. Convergent antibody responses to SARS-CoV-2 in convalescent individuals. Nature. 2020. 10.1038/s41586-020-2456-9 PMC7442695 32555388

[21] de Taeye SW, Bentlage AEH, Mebius MM, Meesters JI, Lissenberg-Thunnissen S, Falck D, et al. FcgammaR Binding and ADCC Activity of Human IgG Allotypes. Front Immunol. 2020;11:740. 10.3389/fimmu.2020.00740 32435243 PMC7218058

[22] Market M, Angka L, Martel AB, Bastin D, Olanubi O, Tennakoon G, et al. Flattening the COVID-19 Curve With Natural Killer Cell Based Immunotherapies. Front Immunol. 2020;11:1512. 10.3389/fimmu.2020.01512 32655581 PMC7324763

[23] Masselli E, Vaccarezza M, Carubbi C, Pozzi G, Presta V, Mirandola P, et al. NK cells: A double edge sword against SARS-CoV-2. Adv Biol Regul. 2020;77:100737. 10.1016/j.jbior.2020.100737 32773100 PMC7292949

[24] Pinto D, Park YJ, Beltramello M, Walls AC, Tortorici MA, Bianchi S, et al. Cross-neutralization of SARS-CoV-2 by a human monoclonal SARS-CoV antibody. Nature. 2020;583(7815):290–5. 10.1038/s41586-020-2349-y 32422645

[25] Yasui F, Kohara M, Kitabatake M, Nishiwaki T, Fujii H, Tateno C, et al. Phagocytic cells contribute to the antibody-mediated elimination of pulmonary-infected SARS coronavirus. Virology. 2014;454–455:157–68. 10.1016/j.virol.2014.02.005 24725942 PMC7111974

[26] Qin C, Zhou L, Hu Z, Zhang S, Yang S, Tao Y, et al. Dysregulation of Immune Response in Patients With Coronavirus 2019 (COVID-19) in Wuhan, China. Clin Infect Dis. 2020;71(15):762–8. 10.1093/cid/ciaa248 32161940 PMC7108125

[27] Zheng M, Gao Y, Wang G, Song G, Liu S, Sun D, et al. Functional exhaustion of antiviral lymphocytes in COVID-19 patients. Cell Mol Immunol. 2020;17(5):533–5. 10.1038/s41423-020-0402-2 32203188 PMC7091858

